# Mechanism of interactions between tripeptide NCW on cellular membrane using molecular dynamic simulation

**DOI:** 10.3389/fnut.2022.1066873

**Published:** 2022-11-03

**Authors:** Sijia Wu, Hong Zhuang, Haiyang Yan, Chen Mao, Bing Wang, Guangdong Zhou, Ge Tian

**Affiliations:** ^1^College of Food Science and Engineering, Jilin University, Changchun, China; ^2^College of Chemistry, Jilin University, Changchun, China

**Keywords:** tripeptide NCW, membrane, absorption, all-atom molecular dynamic simulation, interaction

## Abstract

Tripeptide NCW identified in *Mizuhopecten yessoensis* has been shown to possess *in vivo* antihypertensive effect. However, the poor understanding of the absorption of NCW across the membrane limits its application. In this study, we have investigated the interaction of NCW with DPPC membrane *via* 400 ns all-atom molecular dynamic simulation using GROMACS software. The structural variations of NCW during absorption, the location and distribution of NCW in the membrane, and the effect of NCW on the properties of membranes during simulation were analyzed to understand the dynamic behavior of NCW in DPPC membrane system. The results suggested that the structures of NCW were stable during simulation. Further, NCW could bind on the surface of the DPPC membrane and enter the hydrophobic interior of the DPPC membrane. Residue Try played an important role in the absorption of NCW across the membrane. Hydrogen bonds and hydrophobic interactions stabilized the interaction of NCW with the membrane. All the above studies analyzed the interaction mechanism between NCW and DPPC membranes at the atomic level and laid the foundation for further transmembrane studies of NCW.

## Introduction

Food-derived bioactive peptides have various biological functions and pharmacological effects and exert a positive impact on human health (1). Over the last few years, there has been an increased interest in food-derived bioactive peptides as a promising raw material as health food additives due to their low toxicity, low immunogenicity, high tissue permeability, and easy synthesis and modification (2–4). Absorption in their complete form the gastrointestinal tract and accumulating to a certain amount are essential for the food-derived bioactive peptides to exert any biological activities downstream (5). However, a general lack of understanding of the absorption of these peptides limits their application (1). Peptide-membrane interactions play critical roles in the absorption of peptides across membranes (6). Therefore, the study of the absorption mechanism of food-derived bioactive peptides across the intestinal epithelial cellular membrane is of great significance.

Molecular dynamics (MD) simulation is widely used to study the interactions between peptides and different cell membranes, which is a useful tool to gain insights into the microscopic information about the interactions between peptides and cell membranes, and further understand the mechanism of peptides transport across the lipid bilayer. The all-atom force field is mainly used for the MD simulation of the peptide-membrane system, analyzing the conformational changes of peptide and membrane, and exploring the effect of different phospholipid membranes on the absorption mechanism of peptides. For instance, Ji et al. investigated the interactions between tripeptides, *i.e.*, ADF, FGR, and MIR, and the membrane by using MD simulation. These results demonstrated that hydrogen bonds, electrostatic interactions, and hydrophobic interactions contributed to the binding process for the peptides to the cell membrane, and the positively charged residue Arg contributed to the association of peptide with the membrane (6). The data presented by Lensink et al. demonstrated electrostatic interactions could drive the process of binding peptide penetratin to membranes, and the absorption of peptide penetratin increased the concentration of negatively charged lipids and lipid disorder (7). Furthermore, Pourmousa et al. also used MD simulations to study the penetration of peptide transportan across the lipid bilayer (8). 1,2-Dipalmitoyl-sn-glycerol-3-phosphocholine (DPPC), consists of a hydrophilic head and two hydrophobic tails, is commonly used to study the interaction of peptides with membranes owing to similar properties with natural membrane (9, 10).

In our previous study, tripeptide NCW identified from *Mizuhopecten yessoensis* exerted *in vitro* potent ACE inhibitory activity and *in vivo* antihypertensive activity (11). However, the absorption mechanism of NCW across the cell membrane is still unclear. In the present work, the initial structure of NCW was constructed using UCSF Chimera software. The DPPC phospholipid bilayer structure was constructed through the CHARMM-GUI^1^ website and the tripeptide was placed on the membrane surface to construct the NCW-membrane system. GROMACS software was used to perform 400 ns all-atom molecular dynamics simulations of the NCW-membrane system under isothermal and isobaric conditions to analyze the structural changes of the peptide during the interaction between the NCW and the membrane, to observe the peptide-membrane interaction process and to analyze the influence of the peptide on the membrane structure during the interaction process. The mechanism of the interaction between the NCW and the membrane was investigated at the atomic level.

## Materials and methods

### Structure preparation

The initial structure of the NCW was generated using the UCSF Chimera program (12), and then pre-equilibrated at air-water interface using 100 ns all atom MD simulations with a time step of 2 fs, to obtain the equilibrium and stable tripeptide structure as initial conformation. The simulation was run using CHARMM36 force with TIP3P water model. V-rescale temperature coupling was used for temperature control, and Particle Mesh Ewald (PME) method was used for treating long-range electrostatic. The DPPC bilayers comprised of 128 lipid molecules (64 per leaflet) were constructed using the CHARMM-GUI membrane builder (text footnote 1) (13) and used as the bio-membranes of mammals in this study (9).

### Molecular dynamic simulation of NCW-dipalmitoyl-sn-glycerol-3-phosphocholine membrane system

The pre-equilibrated NCW was randomly inserted into the surface of the DPPC bilayers using the CHARMM-GUI Membrane builder. Then, the NCW–DPPC membrane system was solvated with the TIP3P water model and neutralized by adding a suitable content of Na^+^/Cl^–^ ions. Six equilibration steps at the CHARMM-GUI server were carried out to equilibrate the NCW-DPPC membrane system. The 400 ns atomistic MD simulation was processed by GROMACS 2018 using Charmm36 force field and the TIP3P model of water (6). The simulation was carried out in the isothermal–isobaric (NPT) ensemble and integrated by means of the leapfrog algorithm with a 2 fs time step (14). The temperature was controlled at 323 K using Nose-Hoover thermostat. The pressure was maintained at 1 atm using the Parrinello–Rahman barostat, a coupling constant of 1.0 ps, and a compressibility of 4.5 × 10^–5^ bar^–1^. PME method was used for calculating Coulomb interactions with a cutoff value of 1.2 nm, and the cutoff value of van der Waals interactions was also set at 1.2 nm. LINCS algorithm was carried out to limit the covalent bond (15).

### Stability, compactness, and flexibility analysis of NCW during simulation

Root mean square deviation (RMSD), root mean square fluctuation (RMSF), and radius of gyration (Rg) was calculated separately using gmx_rms, gmx_rmsf, and gmx_gyrate to study the stability, flexibility, and compactness and of NCW, while it was inside the DPPC bilayer. Among them, RMSD was also used to compare the changes in the structure of tripeptide NCW with starting conformation. RMSF was used to measure the flexibility of the residue of NCW during equilibrium, and Rg was used to describe the compactness of NCW (6).

### NCW–DPPC membrane interactions assessing

The center of mass distance change between NCW and the membrane during the simulation was calculated using gmx_distance to analyze the strength of the interaction between NCW and DPPC membrane. The number of hydrogen bonds formed between NCW and DPPC lipids and between NCW and water were calculated by Gmx_hbond. The mass density profile was determined using the gmx_density tool.

### Impact of NCW-DPPC interactions on lipid membrane

The effects of NCW-DPPC interactions on lipid membrane were characterized by bilayer thickness, area per lipid, deuterium order parameter, and lateral diffusion of the membrane using GROMACE built-in tools. The bilayer thickness of DPPC was calculated as the distance between the phosphorous atoms of two monolayers (16).

The area per lipid of the DPPC bilayer was calculated from the cassette area of simulation boxes, *i.e.*, dividing the box area by 64. The deuterium order parameter (S_CD_) for the lipid acyl chain of DPPC was calculated using Gmx_order to analyze the lipid acyl chain stability, and then to illustrate the disturbance of the tripeptide-membrane interaction process on the phospholipid tails. The mean square deviation (MSD) of the DPPC membrane was measured using gmx_msd to analyze the effect of NCW to the lateral diffusion of the bilayer.

### Statistical analysis

The data were analyzed using Origin 2018 (OriginLab, Wellesley Hills, WA, USA).

## Results and discussion

### Peptide structural properties across the cellular membrane

**FIGURE 1:**
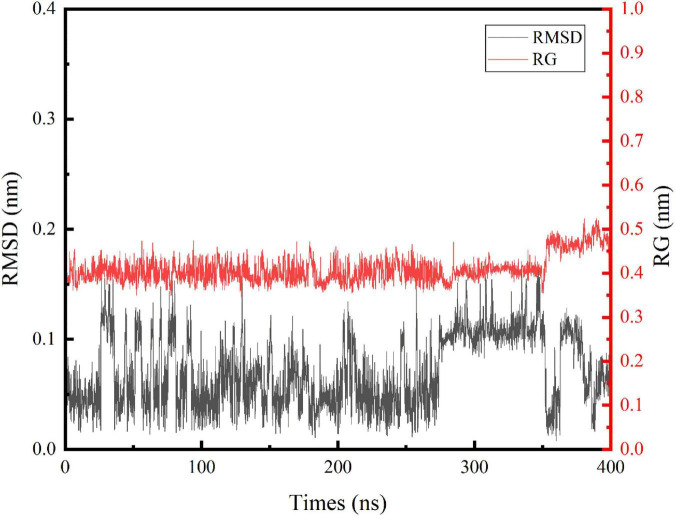
Time evolution of the backbone RMSD and Rg of the tripeptide NCW during the 400 ns MD simulations.

In this study, we performed an MD simulation of the NCW-DPPC membrane system. The RMSD of the α-carbon atoms for NCW (Figure 1) deviated only slightly from the starting conformation throughout the simulation and showed stability oscillating around ∼0.07 Å, which suggested that the simulated system had reached an equilibrium state and the structure of NCW was highly stable in the presence of DPPC membranes. Fluctuations in structures were more obvious for NCW (0.01–0.17 nm) than tripeptide ADF (0.01–0.15 nm) and MIR (0.02–0.14 nm) reported in the previous study by Ji et al. (6), indicating that tripeptide NCW had good flexibility during absorption into the bilayer.

The respective average RMSF in the residues of NCW was calculated to be 0.2368 (Asn), 0.2572 (Cys), and 0.2177 (Trp) during the simulation. Among them, residue Cys seem more flexible throughout the simulation. Furthermore, the local fluctuation of the residues of NCW was largely restricted, indicating a stable molecular structure.

Similarly, changes of Rg values (Figure 1) indicated that NCW maintained a compact architecture during the simulation. The trend of the Rg curve indicated, for NCW-DPPC membrane system, aggregation does not occur easily, which possibly due to the strong interactions between NCW and the DPPC membrane, thereby the movement of the peptide was restricted. Further, the Rg values were slightly increased during 350∼400 ns simulations and then slightly fluctuated until it was stable (around 0.45 nm) for the last 50 ns, probably due to its extended molecular geometry spanning through the membrane.

### Interactions of tripeptide NCW with the cellular membrane

**FIGURE 2:**
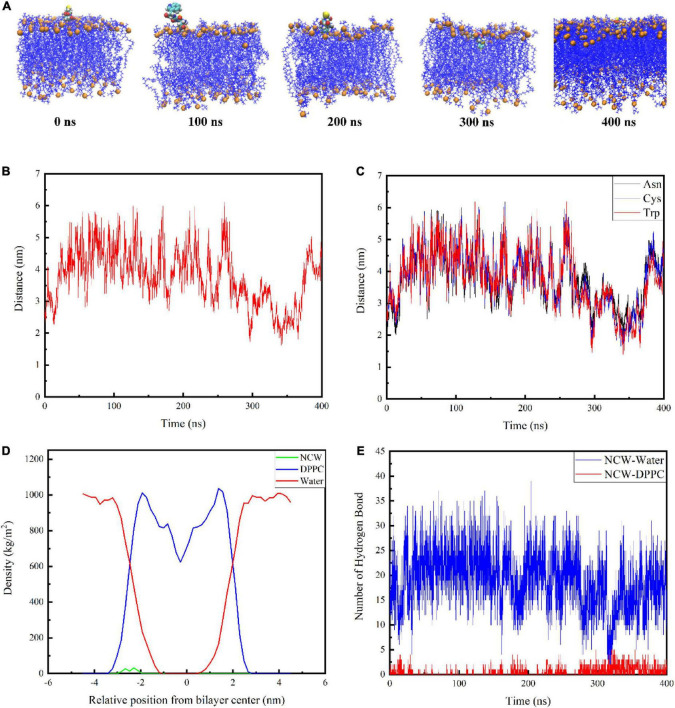
Interactions of tripeptide NCW with the cellular membrane. Snapshots from 400 ns simulation of the complexes formed between the tripeptide NCW and DPPC **(A)**. The center of mass for tripeptide NCW and membrane center distance as a function of time **(B)**. The corresponding mass center distance for each residue of tripeptide NCW with membrane center **(C)**. Mass density profile of tripeptide NCW, water, and DPPC lipid bilayer along the membrane normal averaged from 200 to 400 ns dynamic simulation study **(D)**. The hydrogen bond number formed between tripeptide NCW and membrane, and peptides with water during the simulation **(E)**.

The snapshot conformations of NCW during absorption across the DPPC membrane were shown in Figure 2A. Tripeptide NCW penetrated the DPPC membrane surface and entered the carbonyl terminus of the DPPC membrane. But NCW could not be transported across the DPPC membrane center throughout the simulation.

To further quantify the location of NCW in the DPPC membrane, the corresponding center of mass distance of NCW and the membrane center were calculated and displayed in Figure 2B. And the corresponding mass center distance for each residue of NCW with membrane center was displayed in Figure 2C.

The data presented by Pourmousa et al. showed the distance from the phosphorus atoms mass center of the upper membrane leaflet to the mass center of the membrane is ∼ 2.0 nm, and the distance from the carbonyl groups mass center of the upper membrane leaflet to the mass center of the membrane is ∼ 1.5 nm (8). As shown in Figure 2B, the center of mass of NCW moved toward the center of the bilayer. Tripeptide NCW started to contact the DPPC bilayer surface at ∼290 ns of simulation, and move on the surface of the DPPC bilayer surface. At ∼300 ns, NCW started to contact the carbonyl carbon atom, and subsequently, NCW fluctuated up and down between the phosphorus atom and the carbonyl carbon atom of the cell membrane. We evaluated the distance from the center of the membrane bilayer to each amino acid of NCW to examine further the key amino acids involved in the interaction between NCW and the DPPC bilayer (Figure 2C). It can be seen from Figure 2C, all the residues, *i.e.*, Asn, Cys, and Trp of NCW entered the DPPC membrane. Of these, Trp residue at the C-terminus of NCW was first located closer to the membrane, and interacted with the lipid carbonyl groups of the membrane bilayer, revealing that Trp residue was more likely to be involved in the interaction of NCW with the plasma membrane due to the hydrophobic interactions with DPPC bilayer tail. In summary, the C-terminus of NCW interacted with the cell membrane first.

The mass density profile of NCW along the DPPC bilayer normal was calculated to better characterize their interactions. Figure 2D showed the mass density distribution of NCW, water, and DPPC lipid bilayer along the membrane normal during the last 200 ns dynamic simulation, which well agreed with that was reported before (10). As seen here, the value Z = 0 in the mass density distribution profile corresponded to the central position of the DPPC bilayer. The DPPC lipid bilayer distribution was symmetrical, therefore the bilayer was considered to be stable (17). Moreover, the density of the water is distributed around 1000 kg/m^3^, which corresponds to the actual situation (18). As displayed in the mass density profile, the maximum of the density of NCW was located at approximately 2 nm from the center of membrane. Furthermore, the density of the NCW was centrally distributed within the aqueous phase and the hydrophilic end region of the plasma membrane, suggesting that NCW could interact with the plasma membrane and cross the water-membrane interface, which supported the visual results.

In addition, the number of hydrogen bonds between NCW and the membrane, and between NCW and water, were calculated. As shown in Figure 2E, NCW formed a larger number of hydrogen bonds with water. With the increase in the NCW absorption depth in bilayers, the number of corresponding hydrogen bonds formed between the NCW and water decreased gradually, and finally at about 16. At the same time, the number of hydrogen bonds generated between NCW and DPPC membrane increased gradually. However, the number of hydrogen bonds formed between NCW and DPPC membrane was less than 10. In a word, hydrogen bonds contributed to the binding process of NCW to DPPC membrane.

### Effect of peptides on the structure of cellular membrane

**FIGURE 3:**
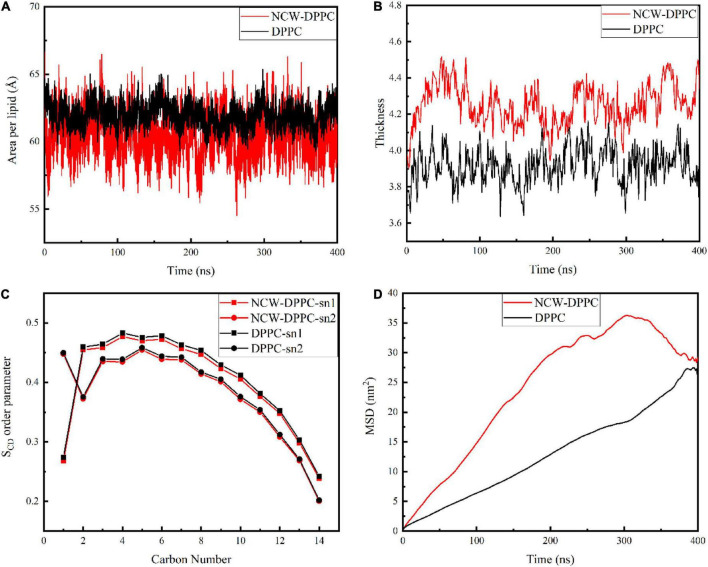
The effect of tripeptide NCW on the thickness **(A)**, area per lipid **(B)**, order parameter of sn1 and sn2 chains **(C)**, and MSD **(D)** of DPPC membrane over the simulation.

Next, the key biophysical properties of the membrane bilayer, *i.e.*, area per lipid, bilayer thickness, S_CD_, and lipid lateral diffusion of the lipid bilayer were analyzed to examine the influence of peptides on DPPC membrane structure during absorption (Figure 3). Both area per lipid and thickness of the DPPC bilayer were critical parameters for evaluating membrane function and stability. As shown in Figure 3A, the average area per lipid for the pure membrane was 61.95 Å, and the average area per lipid value for the NCW-membrane system was 60.41 Å. The area per lipid value slightly decreased when the peptide diffused into the membrane, and the decrease in area per lipid may be likely associated with the ordering of lipid tails for longer-chain lipids (19). Based on above, the insertion of NCW affected the membrane thickness and caused the bilayer to thicken. In addition, no large fluctuations in the area per lipid for the NCW-DPPC system and pure DPPC system were observed, indicating each system had reached equilibrium.

In order to characterize the effect of NCW on the membrane structure, the change in membrane thickness was calculated. As shown in Figure 3B, the average membrane thickness value was 4.25 nm for the NCW-DPPC membrane system over 400 ns, which was higher than that of the pure membrane (3.92 nm).

The S_CD_ was calculated to examine the effect of NCW on the lipid acyl chain (sn1 and sn2) of the DPPC membrane. As shown in Figure 3C, lipid S_CD_ was perturbed upon NCW binding to the DPPC membrane. Tripeptide NCW decreased the S_CD_ from carbons 2-12 and carbons 3-11 for sn1 and sn2 chains of the DPPC lipid, respectively. Particularly, the S_CD_ for carbon atoms close to head groups (C2 to C7) was lower for NCW-DPPC system compared to pure DPPC. Based on these observations, we found NCW increased the lipid chain flexibility, favoring NCW to penetrate the membrane, which might be explained by the subtle hydrogen bond interactions formed between NCW and the membrane. Moreover, NCW led to only a negligible effect on the order of the sn1 and sn2 chains of DPPC compared with the pure membrane, indicating that tripeptide NCW had a weak disruption effect on the membrane.

The MSD values of the phosphate group of the DPPC membrane provided an indication of membrane stability and fluidity. Figure 3D showed the MSD curve of the phosphate group of the DPPC membrane for all systems obtained from 400 ns simulation. The MSD curve for pure membrane system were smooth and seemingly linear in time. An increase in the MSD value for the NCW-membrane was observed, suggesting the lateral diffusion of DPPC was affected by the insertion of NCW, and the characteristic types of motion was observed restricted diffusion mode (20). At the end of the simulation, MSD curve for NCW-DPPC system fluctuated greatly, which possibly caused by the interactions formed between the NCW and the DPPC bilayer tail (21).

## Conclusion

This study simulated the transmembrane absorption of NCW over 400 ns and clarified the molecular interaction mechanism of NCW with the membrane by molecular dynamic simulation. The simulation results indicated that NCW was stably attached to the DPPC membrane surface during the whole simulation. Tripeptide NCW perturbed the membrane, resulting in the increased flexibility, thickness, and lateral diffusion of the membrane. Hydrophobic residue Try could form strong interaction with hydrophobic tail of DPPC membrane, thereby played an important role in the interaction between NCW and DPPC membrane. The transmembrane transport of NCW may be driven by hydrogen bonds and hydrophobic interactions. However, the penetration of NCW through the membrane was not observed in the 400 ns simulation, which may be due to the short simulation time, the composition of phospholipid membrane or other factors. In summary, this work provided the interaction mechanism between NCW and the DPPC membrane at the atomic and molecular levels, and the properties of NCW to penetrate membranes remains to be learned.

## Data availability statement

The original contributions presented in this study are included in the article/supplementary material, further inquiries can be directed to the corresponding author.

## Author contributions

SW: conceptualization, methodology, validation, investigation, formal analysis, data curation, visualization, and writing – original draft. HZ: conceptualization, writing – review and editing, and supervision. HY: validation, project administration, and funding acquisition. CM and BW: data curation and visualization. GZ: writing – review and editing. GT: validation and data curation. All authors contributed to the article and approved the submitted version.

## Footnotes

1
https://charmm-gui.org/
